# Selections and Abstracts

**Published:** 1903-07

**Authors:** 


### SELECTIONS AND ABSTRACTS.

### The Value of the Static Machine as a Therapeutic Agent.*

Whether the use of the static machine should be confined to the development of Roentgen rays, or if the various modalities have any beneficial effect on the diseased conditions to which they are being applied to-day is a question which should be speedily settled.

If there is no therapeutic value in the various applications of the static machine, apart from the development of Roentgen rays, then there is no excuse for the static machine existing, as the coil is cheaper, less bulky and easier of manipulation.

Those who have made a study of the physics and physiology of static electricity give us a long list of diseases in which it is indicated, and insist in no uncertain or wavering terms that cures may be effected where medicine and even surgery have failed.

Before we proceed any further let us dip just a little way into the physics of electricity and see the relationship between the currents generated by a galvanic battery, a faradic battery, and that generated by a modern static machine.

The two chief characteristics of electricity are voltage and amperage. Voltage meaning the speed at which, or potential under which, the current moves; and amperage meaning the size of the current, or current volume. And these are, to some extent, at least the reciprocals of one another. For instance, if we take an electric current of a given voltage and amperage and pass it into a step-up transformer, the voltage of the current is raised, while the amperage is proportionately lowered. And this current may be again passed into a step-down transformer and restored to its former voltage and amperage. There is, of course, a small loss of energy in these transformers in the form of heat.

---

*Read by H. N. Chapman, M.D., St. Louis, Mo., before the Alumni Society of the Medical Department Washington University.

When we consider the electric current as generated by the galvanic battery as ordinarily found in the physician's office, we find a current which usually has a potential of 60 to 100 volts and an amperage of 1.5 to 2 amperes. This current is furnished by 40 to 60 cells connected in series, each giving about 1.5 volts and 1.5 to 2 amperes. When we consider the faradic current we have a current interrupted and induced, and of a comparatively high voltage or potential, several hundred volts and small amperage—few milliamperes; generated by passing the current of 2 to 6 cells (exactly similar to those used in the galvanic battery) into a coil, which is really a step-up transformer, the voltage and amperage varying as the ratio between the winding of primary and secondary coils; an interrupter being introduced into the primary circuit, which interrupter is the essential feature of the faradic battery.

When we come to consider the current generated by a modern static machine, we find that it is an easy thing to obtain 500,000 volts, while the amperage is exceedingly small, perhaps not even 1 milliampere.

So we find a distinct relationship existing between the currents as generated by these three electro-therapeutic appliances. It being an ascending series starting with low voltage and high amperage and ending with very high voltage and low amperage.

The physiological action and therapeutic indications of the currents differ as the voltage and amperage of the currents differ mainly and also as the current is a continuous or an interrupted one.

The chief effect of the galvanic current is a chemical one and this current is used where such an effect is desired; when this current is interrupted it becomes a muscle-contracting one, still, however, manifesting its chemical action to a greater or less extent.

The chief effect of the faradic current is a muscle-contracting one. With a long, fine wire secondary coil, and fine, very rapid interrupter this current becomes a pain-reliever of no mean value, and also a tonic agent not to be despised or lightly set aside.

The static current in its simple administration gives us neither a chemical nor a muscle-contracting effect, yet its effect is a profound one, because we will invariably notice with a patient on the insulated platform of the machine during the passage of the current, a disposition to breathe more deeply, and a marked tendency to perspire; and after fifteen to twenty minutes a tonic sedative effect is distinctly noticeable, and the patient will invariably comment on this latter feature.

Now, the term static as applied to the current from an influence machine is very unfortunate and an entire misnomer. It is very difficult to understand how an electric charge under a potential of 500,000 volts can be at rest. It can not and is not. When a patient is on the insulated platform and connected to a static machine in action we find that a tremendous charge may be communicated to the person, and this charge does not reside on the surface of the body alone. The current distributes itself over and through the body, and as the potential becomes higher and higher it rushes off from all prominences in convective streams, and more continually taking its place. The term static, then, as applied to this apparatus is a misnomer and has prevented many from looking more deeply into the subject. It does not supply a standing charge, but a current flowing over, and in, and through.

The law governing static charges is that a static charge resides on the surface of the body on which it is placed. But it is also an established law that electricity in motion flows not only on the surface but also through the substance of the conductor. We all understand that electricity moves by reason of its voltage or electro-motive force, and it is difficult to understand if 100 volts can cause a current to pass beneath the skin why 500,000 volts can not do it.

What shall we say of the physiological effect of the current from an influence machine? The effects noticed in a patient seated on the insulated platform connected to a machine in motion, are: First, a peculiarly soothing one on the nervous system; a fidgety person becomes quiet and after fifteen or twenty minutes distinctly sleepy. After, perhaps, five minutes there is a distinct inclination for deeper respiration and the patient unconsciously makes full inspiration; further a marked tendency to perspire is manifested.

A number of French writers, of whom we will mention d'Arsonval and Charcot, have investigated the physiological action of the currents from an influence machine. The effects of general electrification are as follows: The pulse is increased in frequency and this increase may persist for even several hours. Arterial tension is increased.

Vigouroux estimates that the temperature is increased about 5° F. d'Arsonval has determined an increase in the respiratory capacity, there being an increase both in the amount of oxygen absorbed and $CO_2$ exhaled. The action of the sweat glands is also increased during the general electrification. There is an increase in the amount of urea excreted in the twenty-four hours, though the volume of urine is not sensibly increased. The digestive functions are accelerated and the appetite improved.

The physiological action of the breeze and spray, the percussive spark and friction spark are clearly defined.

During the administration of these modalities the patient, being on the insulated platform, receives general electrification. With the breeze and spray the effect is on the vasomotor nerves. The capillaries are contracted and the temperature of the skin reduced. During the application of the breeze and spray large quantities of ozone are developed. The reparative processes in skin lesions are increased. The breeze and spray also stop pruritis and calm pain. If resistance is introduced over the parts sprayed, a distinct counterirritant effect is produced which may be carried at will of the operator even as far as vesication. I have seen even the pain of a carbuncle relieved and the patient obtain sleep after fifteen minutes' spraying of the affected part.

The effect of the percussive spark is more profound. When the current which is traversing the body is drawn to one point by the approach of a grounded brass ball and jumps across the intervening air gap in the form of a spark, if in the vicinity of a motor point, a deep contraction of the muscle is obtained. The tissues at this point are subjected to a profound perturbatory action, a contraction of the capillaries at this point is obtained, to be followed in a few minutes by a dilatation, both manifest to the eye. By means of these sparks chronic pains are relieved, old exudations are caused to be absorbed and stiffened joints are loosened up. These are the commoner applications of static electricity, but we have a number of further applications, as, for instance, the static induced current, potential alternation—so-called, Morton wave current and the high frequency high potential currents used with vacuum glass tubes, all of which have their distinct indications and contraindications.

In what class of diseases do we find the static machine of greatest value?

Static electricity is a regulator of functions. Its greatest fields are chronic conditions of malnutrition, and functional and nervous diseases. Neurasthenia and neuralgia, and nervous headaches are rapidly controlled by it; it is of the greatest value in chronic synovitis, rheumatism, chorea, lumbago and sciatica. In certain forms of application it is the most powerful tonic of which we are possessed. A slow convalescence may be materially hastened; insomnia is cured. A man or woman subjected to a severe mental or physical strain may avert the final breakdown by a few properly applied treatments. Schoolgirls at the period of developing into womanhood may be supported and carried through this trying time to their own and their parents' satisfaction; and the woman who is passing through the menopause with all the nervous phenomena we frequently see, and no definite pathological basis for it, may be soothed and comforted and the perverted nervous functions restored to their normal course. Old people in whom the powers of life are waning brighten up wonderfully both mentally and physically under a few treatments.

Can any one repeat such results as have been here indicated? Yes, if they have a machine of proper power, in proper order and know how to use it.

General electrification for every case, or spark, or any other single method used indiscriminately, will not effect results. Each individual case must be studied and the modality best suited applied, and changed as indications point.

What is a proper sized machine?

I would say one capable of giving a good spark, ten to twelve inches long, without Leyden jar connections. For a glass plate machine 8 30″ revolving plates. For a mica plate machine 4 28″ or 30″ plates, but revolving at a more rapid rate of speed than the glass plate machine. Nothing less than this is capable of giving permanent therapeutic results. My own machine has 16 32″ revolving plates and 16 stationary plates, and will, under the best conditions, give a fat spark fifteen inches long.

Just a word as to the static machines belonging to suggestive therapeutics. I have used a static machine now for two years; for one and a half years an 8 30″ revolving plate machine and for the past six months my larger apparatus of 16 32″ revolving plates, and my profound conviction is that there is no more suggestive therapeutics about static electricity from a machine of adequate power, properly handled, than there is about any reputable procedure of medicine or surgery.

Let me detail a case recently treated:

H. C., aged fifty-five years, became a confirmed neurasthenic two years ago, with frequent suicidal inclinations, especially desiring to kill himself with a shotgun. He had traveled seeking health, and during the past eighteen months had been treated by one of our best general practitioners. He was urged by his friends to come and take electrical treatment but was prejudiced against it and refused. Finally, to relieve himself of their solicitations he spoke to his physician about it, and was informed that electricity was not suited to his case, and under no circumstances to take it. This silenced his friends and the matter was allowed to drop for six months. At that time, a little over a month ago, I was called to see him. I found a man, elderly in appearance, of spare habit, in bed, where he had been for twenty-four hours, languid, falling away into a dozing condition, but easily aroused. The pulse was rather slower than normal, temperature normal. The heart and lungs gave negative results; the tongue was furred, bowels constipated, skin dry; appetite variable, digestion not good. The slightest mental or physical exertion caused his mind to become chaotic and he could not then concentrate his attention on the subject in hand.

He wanted to know what electricity would do for him. I told him to come and see me and I would ask him to give me a trial every day for one week and then three times a week for three months, and if at the end of that time he was not materially improved I would not continue treatment. He was not able to get out for a week after I saw him and then he came.

I began treatment and pursued it vigorously (using the wave current applied over the epigastrium or lumbar and sacral, or cervical and upper dorsal), and in four weeks, in all about fourteen treatments, he has been enabled to go back into his office and take up his business, that of a title examiner. Every function has been restored. He has an appetite, as he says, like a horse; digestion normal, bowels perfectly regular and the stools normal in appearance. He sleeps soundly all night and wakes refreshed. His mind is alert and active; grasps the problems of a title and no sense of confusion or mind weariness.

Last Monday, just four weeks from commencing of treatment, he went to the court-house and worked from 8 a.m. until 2 p.m. over a very difficult title, apprehensive that he could not complete it, finished it, went to his office and worked until 5 p.m., writing it up, ate supper, went to bed at 9:30 p.m. and slept, as he said, like a babe all night, awoke in the morning and reached his desk at 8 o'clock. The only inconvenience he felt was a slight muscular soreness; but remember that this was the first day's work at his desk for two years.

One curious phenomenon occurred after the fifth or sixth treatment: The patient had not perspired for a year in a normal way, and not at all for months, even after physical exertion, but just at this time, a warm day occurring, he worked in the garden and perspired freely, which surprised him very much. That night, after a bath, he "shed his skin," as he expressed it. The epidermis peeled off in such quantities and such large pieces that it blocked the opening in the bath tub as the water ran off.

It would be difficult, indeed, to attribute this result of static treatment to suggestion. It was not suggestion for I promised him nothing, and he came because his wife insisted upon it. He is practically a perfectly restored man. There is nothing in the whole range of therapeutics, to my knowledge, but static electricity that would have accomplished it.—*Courier of Medicine.*

### URETHRAL STRICTURE.*

Though not attempting to enlarge the general fund of knowledge concerning stricture of the male urethra, I will briefly discuss certain phases in its treatment. Gonorrhea is, as we all know, most frequently the exciting cause, still among other conditions that figure in the etiology of stricture may be mentioned alcoholism, traumatism and sexual excesses. Sometimes in diabetes or in the uric acid diathesis, where the urine is excessively irritating, stricture eventually results; and during the recent bicycle fad a few years ago, it was not an uncommon occurrence for a stricture to be directly traceable to the persistent riding of a bicycle with a saddle, the convex surface of which exerted undue pressure upon the perineum. Then too, in the treatment of gonorrhea, many strictures are due to the use of too strong injections, or even to the employment of weaker ones during the acute stage of the inflammation. In fact anything tending to produce chronic irritation and congestion of the parts is liable, sooner or later, to result in stricture.

Like any other form of chronic inflammation, urethral stricture begins with granulations and develops into cicatricial tissue, which, by continued contraction, tends to narrow if not to obliterate the lumen of the urethra. Being a proliferation of mesoblastic elements, the granulations and cicatricial tissue may sometimes be interspersed with other mesoblastic elements, as cartilage and elastic tissue. Such is in brief the pathology of organic stricture, but there are also strictures of a purely functional character excited by the inflammation accompanying organic strictures. These functional strictures, reflex spasms of the compressor urethræ muscle, are frequently a great obstacle in the treatment of organic strictures of small caliber.

Strictures of the urethra occur most frequently either near the meatus or just anterior to the triangular ligament. Further back, for instance seven inches or more from the meatus, an obstruction may more properly be considered as due to an enlarged prostate,

*Read by F. D. Patterson, M.D., Mentor, Minn., before the Red River Valley District and County Medical Society at Crookston, April 28, 1903.

and rectal examination will readily confirm the diagnosis. Strictures may be long or short and may occur either singly or two or more in the same urethra.

However caused, stricture, if neglected or improperly treated, tends to diminish in caliber, the inflammation extending backward into the bladder causing cystitis. Back of the stricture the urethra becomes overdistended. The bladder becomes hypertrophied, its walls thickened, and in consequence of back pressure, sacculated. From deficient outlet, the urine accumulates, stagnates and assumes an alkaline reaction, thereby favoring the formation of phosphatic calculi. The inflammation may eventually extend up the ureters and involve the kidneys, causing suppurative pyelitis and nephritis, a condition frequently spoken of as surgical kidney.

The patient usually complains of frequent and painful urination with but a few drops passing at each time, the stream being considerably narrowed; yet before the pathological conditions incident to stricture have very extensively developed, the stream is frequently full, and the patient, previous to the passage of sounds, has not the slightest suspicion as to the existence of stricture. There is usually a thin mucous discharge gluing the lips of the meatus together. The urine is of a cloudy consistency, of low specific gravity, there usually being fine mucous sediment present but the absence of clap-shreds. Frequently the reaction is neutral or alkaline. In severe cases, the abdominal muscles are brought into play to assist the muscles of the bladder in the attempt at emptying that viscus, and the violent efforts at thus straining sometimes result in piles, prostatic congestion and occasionally in prolapse of the rectum. Besides these complications, neuralgia and other reflex neuroses sometimes result.

In an ordinary case of stricture, it is a simple matter, after sterilizing the sound and the patient's urethra and warming and lubricating the sound to place the patient in the lithotomy position, raise and elongate the penis, gliding the sound along the floor of the urethra until the triangular ligament is reached, then depressing the penis, letting the sound glide along the roof of the urethra and by its own weight enter the bladder.

In the average stricture of not too small caliber nor of too great sensitiveness, three different sounds can usually be passed at each sitting without much danger either from shock or from urethral fever. After passing a sound into the bladder, it should, with a view of stretching the parts, be left in about five minutes before passing the next sound. This is, in the American scale, usually two sizes larger than the first. If there should occur a spasm of the compressor urethræ or too free hemorrhage, the passage of instruments at that sitting should cease and no further attempts at catheterization should be made for at least two or three days. Where great tenderness exists, it is sometimes desirable, before attempting to pass any instruments, to make a local application to the urethral mucous membrane of a one per cent. cocain solution, of course bearing in mind the greater danger of mechanical injury to the parts in consequence of their sensibility being thus somewhat deadened. In simple cases, frequently two or three sittings for the passage of sounds are all that are required.

The extent to which the urethra must eventually be dilated is most conveniently formulated by Otis's rule, which is as follows: A penis three inches in circumference requires a No. 30 sound, French scale, and every additional $\frac{1}{8}$ inch of penile circumference increases the size of the required sound by one French number. Thus a $3\frac{1}{8}$ inch circumference requires a No. 31 sound, $3\frac{1}{4}$ inch No. 32, and so on.

In numbering the sizes of catheters and sounds, much needless confusion arises because there is in use more than one scale of measurement. The two scales in most common use are the French and the American. In the French scale, the unit of measurement is one number for every 1-3 millimeter in diameter, thus No. 1 is in diameter 1-3 millimeter. No. 2, 2-3 millimeter, No 3, 1 millimeter, and so on. In the American scale, No 1 is $\frac{1}{2}$ millimeter in diameter, and each additional number represents an additional increase in diameter of $\frac{1}{2}$ millimeter. From these figures, it is an easy matter to reduce numbers from one scale into the other. Thus Otis uses a No. 30 French size to fit a penis three inches in circumference, which therefore is, in the American scale, a No. 20 sound.

Besides the passage of sounds, the use of soluble bougies of the following composition has, in my experience, proved a valuable adjunct:

Tanic acid,

Boric acid,

Ichthyol......................................aa ½ gr.

Balsam of peru ....................................¼ gr.

Oleate of zinc.......................................⅙ gr.

Cacao butter, q. s.

The patient before retiring and immediately after urination, inserts one of these soluble bougies up the meatus every other night, or every night, according to the urgency of the case. The general health should also be looked after and careful attention given to the action of the bowels. In this class of cases I have sometimes prescribed for internal administration various combinations of some of the following: saw palmetto, sandalwood, copaiba, tincture of cantharides, buchu, hydrastis and other genito-urinary stimulants and tonics, but of late am coming more to the conclusion that such internal medication, in cases of stricture, is totally unnecessary, even though it has some merit in the treatment of gonorrhea and cystitis. On the whole, it tends to irritate the stomach, thereby indirectly aggravating existing conditions. While undergoing treatment for stricture, the patient should abstain most rigidly from too stimulating diet, alcoholic stimulants and sexual excitement; for, as in other forms of inflammation, rest, together with the avoidance of irritants, is of the utmost importance.

Having briefly discussed strictures readily penetrable by ordinary steel sounds, I will say a few words concerning such strictures as, only with the greatest difficulty, even so much as admit a filiform bougie. Unless special care is taken to relax the spasmodic condition, it is utterly impossible to introduce through the stricture into the bladder any instrument whatever ; but even in these tight strictures, Otis's rule can, in almost every instance, be eventually complied with without having to resort to internal or to external urethrotomy. In order to relax the spasm of the compressor urethræ which attends all deep strictures of very small caliber, the patient must first be comfortably and thoroughly warmed, and no attempt at sounding the bladder should be made in a cold room.

Also $\frac{1}{8}$ to $\frac{1}{4}$ grain of morphine should be injected into the perineum, or in extreme cases, general anesthesia may be employed; but owing to a possibility that the kidneys may not be in a healthy condition, it is better to dispense with general anesthetics, especially ether. All instruments passed up the urethra must be thoroughly warmed, for a cold sound applied to an oversensitive urethral mucous membrane will invariably produce a spasm which will prevent for a long time the introduction of sounds into the bladder. In order to avoid a reflex spasm, the greatest gentleness must be used, and even then, with all these precautions taken, the spasm will invariably, for the first few times, prevent very extensive manipulation of sounds at each sitting.

The bladder should not be evacuated too suddenly, lest, by relieving the greater abdominal vessels of that pressure before they have time to recover their tone, they become engorged, leaving the upper extremity in an anemic condition, thereby tending to cause stoppage of the heart from deficiency of blood.

It must also be borne in mind that the smaller the diameter of the sound, the greater is the danger of making a false passage; hence in strictures of small caliber, the more imperative it is that, without a guide, no force whatever should be used in entering the bladder. Should, however, that accident occur, it may readily be detected by sudden deflection of the sound from the median line, sudden manifestation of pain on the part of the patient as well as sudden hemorrhage. Should the instrument be a hollow catheter, the fact that no urine escapes would also be an indication that the instrument was in a false passage, and this can be still further verified by rectal examination. Should a false passage occur, no further attempts at catheterization should be made for at least ten days.

After thorough relaxation of the parts, several filiform bougies are introduced through the meatus, and by careful manipulation, one at least can usually be made to enter the bladder. A sound grooved on its convex surface with an eye at its extremity on which to thread the filiform bougie is then threaded onto the projecting end of the bougie that was passed into the bladder, and with the bougie as a guide, the grooved sound can be forced into the bladder without danger of making a false passage. The lips on the convex surface serve in spreading apart the granulations and cicatricial tissue. Great care must be taken, while so doing, to firmly hold the projecting end of the filiform bougie, for in letting that slip out of one's grasp, the bougie is liable to be forced so far up the urethra that to extract in would require a perineal section.

Sometimes in strictures of small caliber, it is just as well to leave the filiform bougie *in situ* twenty-four hours, when sufficient softening of the tissues will usually have taken place to allow the passage of a small catheter. While an instrument is thus retained in the urethra, the urine should be rendered aseptic by the internal administration of from five to ten grains of salol three times a day.

After penetration of the stricture by the grooved sound, in almost every instance a steel sound No. 12 of the American scale can readily be passed, and frequently larger sizes. Occasionally the grooved sound with a filiform bougie as a guide, has to be passed at two different sittings, but usually not. If there is not too great tenderness, sounds should be passed at two or three sittings a week, and after the normal caliber of the urethra has been reached, the sized sound indicated by Otis's rule should be passed once a week for three weeks. For a while, previous to the passage of instruments, morphine should be injected into the perineum, but later on the perineal injection should be omitted. When, with the omission of this injection of morphine, a steel sound of the normal capacity of the urethra can be passed without setting up a spasmodic stricture, the patient may be considered as practically cured. Finally to guard against a recurrence of the stricture, the patient should be instructed in the use of a large soft rubber catheter, No. 32 French for instance, and pass that every day for three months.

Anterior strictures are, as a rule, considerably more resistant to treatment than are posterior. This fact is readily accounted for by the greater elasticity of the penis as compared with the bulb, yet even those strictures will usually, by persistent dilatation, either by divulsion or by the passage of sounds, in time become dilated to the normal caliber of the urethra.

While not denying that in extreme cases, internal and external urethrotomy might sometimes be indicated, still, judging from the results obtained by dilatation, even in strictures of the smallest caliber, one has good reasons for believing that, in many instances, these operations are performed when totally unwarranted.

Under what circumstances, then, should resort be made to operative treatment? For anterior strictures of such an indurated character as greatly to resist intermittent or continuous dilatation, or for elastic strictures which, though admitting fair-sized sounds, immediately close up after the withdrawal of the same, internal urethrotomy is the operation indicated.

Only in strictures anterior to the scrotum, on account of the elasticity of the penis, can internal urethrotomy be performed with safety. Further back, this operation is frequently followed by extravasation of urine, cystitis, septic infection, severe hemorrhage, pyelitis and nephritis.

When a posterior stricture is totally impervious to instruments, external urethrotomy may be performed; but, as a general rule, are there any advantages in this operation not obtainable in the use of bougies? In the first place the old method of perineal section without a guide is far from satisfactory, for the urethra is much more difficult to find than might be inferred from the illustrations in Gray's Anatomy. Sometimes in impervious strictures with posterior dilatation, what is known as Cock's operation may be performed. This consists in tapping the dilated portion, with the finger in the rectum for a guide, the object being that by relief from the pressure and the irritation of the urine, the stricture may eventually become pervious. However, it is the rarest kind of an exception for a stricture to exist that will not, sooner or later, admit a bougie. In the Syme's operation and its various modifications a staff must be introduced as a guide clear up into the bladder, and in the Wheelhouse operation the staff must reach down to the deep stricture, penetrating any interior stricture that may exist. If then the urethra is capable of admitting a guide for external urethrotomy, would it not be much more reasonable to insert, instead of the staff, a bougie, and by patient perseverance gradually increase the size of the sounds passed until the urethra is dilated up to its normal caliber?—*St. Paul Medical Journal.*

### DRUG TREATMENT FOR TUBERCULOSIS.*

No one will admit to-day that there is a specific drug treatment for tuberculosis, but this is far from saying that we have no useful drugs. To-day as never before the attitude of the medical profession toward the "white plague" is that of hope, for already the ratio of cures has greatly increased and is increasing yearly. Thousands of physicians are absorbed in a search for a cure, and success must attend their combined efforts.

The purpose of such evenings as this is to bring in review the results of these various investigations, in order that we may select from the mass that which seems good. If we then utilize in our private practice this new knowledge, and keep an accurate record of our cases, we add materially to the total sum of knowledge.

All are agreed that the ideal treatment of tuberculosis consists in a happy combination of climate, diet, exercise, rest and medicine. To the latter I will now direct your attention for a brief summary, discussing only those that appear the most helpful. For years a specific was sought for in the inhalations of various antiseptic drugs, such as carbolic acid, sodium benzoate and iodoform. Each in turn was enthusiastically recommended and then discarded. More recently the inhalation of pure oxygen, also formaldehyde, has been brought to our attention. Trudeau and others after many experiments have shown that these antiseptic substances do not penetrate sufficiently into the diseased areas, and hence their failure. But, unquestionably, they are a help in the beginning cases. Oxygen should not be used where there is a tendency to hemorrhage, as it is extremely irritating.

Internally arsenic pills, gr. one-twentieth, three or four times a day has been much lauded, but it has little influence except where there is anemia, etc., in the earlier stages. Sodium cacodylate has replaced it. Next came creosote, and in doses of from fifteen to thirty m. in twenty-four hours it has proven very useful. Everyone is familiar with the wide discussion of this drug, and as an illustration of how widely used it is I will quote Dr. J. A. Burroughs whose records show 2183 cases collected during the past

*Read by Lester W. Day, M.D., Minneapolis, before the Minneapolis Medical Club, April 20, 1903.

nineteen years, and of these 1315 had previously taken or at the time of his examination were taking creosote. The drug is useful because it is eliminated by the lungs and kidneys, and also because it is a tonic. But it is extremely disagreeable to take, and is also irritating to the stomach. It has been supplanted largely by guaiacol, the essential principle, given as guaiacol carbonate in powders. The dose should be gradually increased to thirty-five or forty grains a day. The drug has little of the disagreeable taste and odor of creosote, but is much more expensive. Guaiacol is of especial advantage where there is purulent expectoration.

Landerer, chief of the Institute of Knihenbad in the Black Forest, published his first monograph upon the use of sodium cinnamate, or hetol, in 1892. Only recently has it received the attention that it deserves.

The principle upon which the treatment is based is as follows: So long as inflammatory changes in a lung do not progress beyond the stage of hyperemia or serous exudation complete restitution is possible; but where necrosis has occurred the most favorable conclusion is the formation of a scar. In tuberculosis in the majority of instances necrosis has occurred when we first see the patient, and our object is to produce a scarification. But it is well known that tubercular areas have comparatively little tendency to scarify, but rather to break down and form chronic abscesses, fistulous ulcerations, cavities, etc. Landerer attributes this characteristic to the very moderate inflammatory reaction in and about tuberculous areas, and he points in confirmation of his views to the marked scarcity of blood-vessels in them. Hence he conceived the idea of provoking a more active inflammation about the areas. Foreign particles injected into the blood are taken up by the leucocytes and deposited by preference in the regions where an inflammation is present. While experimenting upon rabbits with an emulsion of balsam of Peru he found that after an intravenous injection of the balsam there was an active leucocytosis, which reached its height in from three to eight hours. These leucocytes containing fine globules of balsam were found dilating and crowding the capillaries about the diseased areas. In the course of a few weeks, and after repeated injections, he found granulation tissue springing up and extending into the substance of the tubercular area gradually absorbing and replacing the necrotic material of which it is composed with scar tissue. Cinnamic acid is the efficient constituent of balsam of Peru, and the sodium salt, otherwise named hetol, is the substance used for the intravenous injections. The injections are entirely harmless, and even excessive doses cause no discoverable injury to any organ. However, when given in excess the patients complain of great drowsiness.

Whether the benefit is due to the leucocytes or, as Landerer suggests, hetol acts on tuberculous processes by forming innoxious compounds with the toxins of the tubercle-bacillus, has not been determined. Landerer disclaims any specific action for the drug, and is of the opinion that purely tubercular infections are the only ones benefited. He begins with a dose of from 1-100 to 1-50 of a grain, and gradually increases the dose up to 1-4 or 1-3 of a grain. One injection should be given every other day. His good results have been verified by many competent observers.

Henry Harper, in a series of papers appearing in the *Lancet* (London) in 1901 and 1902, points out the great benefits derived from the ingestion of large doses of pure urea into the system. He was led to experiment along this line by the consideration of the fact that families of marked gouty tendencies rarely if ever had tuberculosis; that an attack of gout in the midst of tuberculosis caused the disease to stand in abeyance; that tubercular patients were rarely meat-eaters; and that the tubercle-bacillus would not grow in a medium containing nitrogen. Cautiously he began to experiment. He found his patients would take 30, 40, 60 grains of urea three or four times a day without injury, and, moreover, without increasing their nitrogenous excretions or secretions. In other words the urea is utilized in the system. Moreover, the patients rapidly gained in weight, and there was a rapid amelioration of their general condition. Personally, I have followed his plan during the past year with very satisfactory results in a number of cases.

It is a suggestive fact that during pregnancy when the woman's system contains an excess of the products of catabolism tubercular processes are usually held in abeyance. The explanation advanced is that these substances act as an antitoxin. Now, are there other antitoxins? Richet and Hericourt have proven that the juice of raw meat acts, not as a strengthening agent, but as an antitoxin. At Cannes the patients who are able eat as much as they can of twenty-eight ounces of mashed raw meat; the remainder is pressed, and they drink the juice. This diet has many advocates, but it is expensive, and is, moreover, supported with difficulty by many patients.

The medical literature from 1890 to 1895 shows that the blood of certain animals confers on other animals immunity from tuberculosis, and may even cure this disease. Berlin and Picq have introduced a serum (Goat's serum) which some at least of the French physicians believe to be tonic, antitoxic and bactericidal. The treatment is innocuous and easily applied, 2 c. c. being given every other day hypodermically, or 10 c. c. if given by mouth.

There are countless other drugs that have been and are being tried whose merits and demerits I have not taken up. The failure of any one plan or combination of treatments to cure tuberculosis is easily understood when we consider the various modes of attack of tuberculosis. Most of the cases reported cured are the early cases, that is, the cases coming under treatment before the action of the t. b. has been enhanced by that of the staphylococcus, and the streptococcus, also before the body has lost its power of resistance.

We should be careful, in trying the so-called specific drugs, not to lose sight of our general symptomatic line of treatment.—*N. W. Lancet.*

---

### THE IMPORTANCE OF THE EARLY RECOGNITION OF APPENDICITIS.*

The vermiform appendix and the varied pathological conditions which it so often presents will be subjects for discussion by medical men long after we have fulfilled our mission as physicians and surgeons.

There is no reason to believe that the environment of modern

*Read by Clarence L. Wheaton, M.D., Assistant in Gynecology, Denver and Gross College of Medicine, Denver, Col., before the Denver and Arapahoe Medical Society, December 16, 1902.

civilization creates any predisposition to appendicitis. Our forefathers doubtless suffered with the disease, and, in many instances, died during an acute attack, after rupture of the appendix and the onset of a general peritonitis, then diagnosed as inflammation of the bowels. In late years the careful taking of clinical histories, the improved methods of technique in diagnosis, and our training to observe all minor details in the presence of disease, have been the essential factors in contributing so much to our knowledge of appendicitis.

The literature on this subject is most exhaustive and we find many differences of opinion concerning the etiology and treatment of the disease. Many reputable men have endeavored to solve the mystery as to the function of the appendix, but we are prone to be skeptical regarding their theories.

Notwithstanding all this, we have gleaned facts from a great army of workers in this field of medicine and surgery. We know of the surgeon in the small country town who has had the courage to operate on his cases under the most trying circumstances, thereby contributing something of value to the surgery of appendicitis. We are familiar with the work of the general practitioner of medicine who has many times successfully carried his patients through acute attacks of the disease without surgical intervention, thereby contributing facts of value to "internal medicine."

It is recorded in the surgical history of appendicitis that W. W. Grant ligated the appendix January 4, 1885, that T. K. Morton removed the appendix February 21, 1887, and that C. B. Stemen performed an appendicectomy April 22, 1887.

In conversation with Dr. W. W. Grant relative to the early operations for appendicitis, he kindly gave me the following history of the case he operated on in 1885, the earliest recorded operation for appendicitis :

"On January 4, 1885, I cut immediately down to the cecum, opening the peritoneal cavity and searching for the appendix. It could neither be felt nor seen, but from the position and attachments of the cecum I was satisfied the appendix was embedded in the iliac fossa. It was thoroughly excluded from the peritoneal cavity. By breaking down the diseased tissue to the right of the cecum and outside the peritoneal cavity with the index-finger I found the appendix and ligated and removed it; wound drained with iodoform gauze and abdomen closed without drainage. A fecal fistula followed which was operated and closed January 26, 1892.

"Patient at the present writing alive and well."

It should be a source of pride to every American physician to know that his countrymen have been leaders in this field of medicine and surgery. We have profited by the investigations of these men, and we have been able to glean facts which if properly applied enable us in the majority of cases to successfully combat the disease.

It is not my purpose in presenting this paper to allude to any methods of surgical technique nor in any way elaborate upon the treatment of appendicitis. I shall, however, call your attention to the varieties of appendicitis and the importance of their early recognition in furnishing the indications for proper treatment.

In the catarrhal type the pain radiates throughout the abdominal region, being intense at the epigastrium, finally becoming localized to McBurney's point, where tenderness only may be elicited on pressure. The rectus muscle on the right side is usually tense, this condition gradually disappearing as the acute symptoms subside. Temperature is usually 103° to 104°, with a pulse in proportion, nausea and vomiting present. The duration of the attack is from two to four days. In these cases we find upon macroscopic examination that the mucosa is congested and the lumen of the appendix obliterated at several points.

The erosion of the muscosa following this type of bacterial invasion exposes the submucous tissue to infection and to future inflammation.

It is this type of the disease in those cases where operation is refused that run a chronic course and not infrequently resolve themselves into the so-called suppurative type of the disease. These attacks may be sudden in their onset, with severe pain, the nausea and vomiting becoming distressing, with a temperature of 104° or higher, and rapid pulse; tenderness is early felt at McBurney's point, and rigidity of the abdominal muscles is very marked, especially on the right side over the site of the cecum. The symptoms are similar to those of the catarrhal type of the disease but in every respect more severe and lasting five or six days. The appendix in these cases rapidly fills with pus, and at times becomes a purulent sac. Contiguous peritonitis with adhesions may follow this type of the disease and a tumor mass be felt upon the right side over the cecum. Should the disease be of sufficient duration the so-called peri-appendicial abscess may follow and its advancing walls, as the circumference of the tumor mass increases, carry infection to remote regions. The fatal termination of these cases may result only after a long illness, through septicemia, or the abscess may rupture into the bowel or become encapsulated and absorbed. The sudden fatal termination of these cases is usually due to rupture into the general peritoneal cavity, which speedily closes the scene.

In the perforative type of appendicitis the obliterated lumen becomes overdistended and ruptures at the point of least resistance, which is usually a necrosed or ulcerated area. Localized gangrene following the impaction of an enterolith may so weaken the wall of the appendix that it ruptures. Finally muscular exertion may cause a rupture of the appendix, but these cases are comparatively rare.

If the duration of the suppurative type of appendicitis has been sufficient to have fortified the general cavity by adhesions, the disease, notwithstanding the rupture, may take on the suppurative type of the disorder already mentioned. On the other hand, if there are no limiting adhesions, violent pain, collapse, fearful abdominal rigidity and septic peritonitis soon terminate the case.

The gangrenous type of the disease is fortunately rare. It is here proper to allude to the chief source of blood supply to the appendix which is a branch that arises from a loop of the colica media. This branch runs along the border of the meso-appendix, giving off branches which pass into the organ. When the meso-appendix is wanting, the artery runs directly along the peritoneal covering of the appendix. In exceptional cases, according to Fowler, the vessel passes directly to the tip of the organ, giving off no beanches until it is reflected in the submucosa. It is in these cases of limited arterial supply that rapid gangrene occurs.

In the gangrenous form of appendicitis, endarteritis and thrombosis of the blood-vessels must be considered as causative factors following a suppurative destructive process, producing a rapid necrosis of the gut. A profound septicemia is always present in these cases, with early collapse and rapid dissolution. The so-called chronic type of the disease is merely relapsing or a recurrence of the types of the disease already mentioned, the patient being fortunate enough to have survived the primary attack.

Such I believe to be the conditions as they present themselves at the operating-table and at autopsy, and I believe that great importance should be attached to their early recognition as furnishing the indications for treatment.

Little difficulty would perhaps be encountered in recognizing the advanced types of the disease, such as gangrenous and perforative appendicitis, but we must not scoff at the man who even fails here, as I have known the ablest of surgeons to open the belly and find other conditions than those diagnosed. The suppurative and catarrhal types, however, present the greatest difficulty as to diagnosis, and no doubt have baffled many men within hearing of my voice. The congested and enlarged appendix cannot be easily palpated through the abdominal wall, and I am in doubt as to whether we can be positive that we recognize the organ on palpation. Especially liable are we to err in females owing to other structures in the vicinity of the appendix.

Pulse, temperature, abdominal rigidity and facial expression are important factors from which to argue existing conditions, the increase or diminution of these taken as signs showing an increase or diminution of the destructive process. A slight catarrhal attack may have more of deviation in it than the severe suppurative form of the disease. We have seen gangrene of the appendix with gangrene of neighboring organs and with no clinical history suggestive of a formidable change.

Treves says that 20 per cent. of all post-mortems reveal past appendicitis. It seems almost incredible that these cases, like peaceful nations, should have made no history.

In the light of our knowledge of recurrent attacks of the disease in many patients, some resulting in death, I believe that he who operates the most at the proper time is right. Patients do not die from the effects of appendicitis operations performed by competent surgeons. It is the disease that destroys life. I am to-day more than ever convinced that early surgical intervention would save life in nearly every case.

The story of appendicitis never has been written and never will be, so that he who runs may read. The man who would solve the problem presented by this disease in the sick chamber must possess those attributes of learning gained by hard work and experience that will justify us in calling him an able physician.—*Colorado Medical Journal.*

---

### AMEBIC DYSENTERY.*

I have no intention of taking up your time with an elaborate presentation of either the historical review, pathology or morbid anatomy of this disease. I only intend a brief résumé of the salient points about the disease, and some clinical notes on cases recently seen in the wards of the United States Marine Hospital at Louisville.

In the beginning, I announce my acceptance of the doctrine that the disease is a specific one, differing in causation, morbid anatomy, and to some extent in clinical aspects from other dysenteric disorders, such as the pseudo-dysentery which is often only the consequence of a very severe ileocolitis or proctitis, and the dysentery of bacillary origin; or those cases of bloody diarrhea sometimes seen in extremely malarial sections, which yield promptly to the administration of quinine by the mouth, are accompanied by the presence of estivo-autumnal parasites in the blood, and which, I permit myself the digression to observe, are probably due to the localization of large numbers of parasites in the capillaries of the villi. The statement of this belief obviates all necessity for a résumé of the views of the different investigators of the subject, or of a discussion of the observations upon which these views are

*Read by G. B. Young, M.D., Past Assistant Surgeon, U. S. Public Health and Hospital Service, before the Louisville Clinical Society, March 31, 1903.

predicated. As my remarks upon the disease are intended mainly as an introduction to the report of cases observed here, I will restrict them to a summary.

The cause, then, is an ameba. It seems well established that there are several amebæ inhabiting at times the large intestine, and sometimes confounded with the true ameba dysenterica, but this latter has characteristics which serve to differentiate it from its fellows. It is a clear body, four or five times the size of a red blood-corpuscle, with a very faint greenish tinge while alive and active, and grayer and duller looking when dead. The ectosarc is clear and the endosarc granular, with usually a few vacuoles. When there is much blood in the stools it nearly always contains several blood-corpuscles. As long as the examined material is kept warm it is quite active, but rapidly becomes motionless when allowed to cool.

*Mode of Infection.*—The overwhelming mass of evidence accumulated during the past few years leaves no doubt that the disease is primarily water-borne. There are other methods of infection, but they account for only a fraction of the total cases. Handling infectious material is probably the most frequent of the secondary causes.

*Predisposing Causes.*—Briefly, these may be any of the mental, functional, pathological, or even meteorological conditions which unfavorably affect the powers of resistance. The most frequent, however, is the occurrence of intestinal derangements of the kinds apt to produce lesions of the mucous membrane, as catarrhal inflammations of either digestive or bacterial origin.

*Pathology.*—The characteristic intestinal lesions are small, lineal ulcerations in the colon, especially in the lower part of sigmoid or in the rectum, apparently following the line of blood-vessels or lymphatics, crossing each other at places like worms' tracks under a tree's bark, extending down into and through the submucosa, and invading the latter to a greater extent than the other coats. From this comparatively simple lesion up to extensive gangrenous sloughs there are ulcerations of all shades of shape, size and intensity. It seems probable, however, that most of these latter conditions are the result of secondary infections, as with the coli communis, or the various pyogenetic bacteria.

*Sequelœ.*—The most common characteristic of these, so common indeed that it is a question whether it should be classed as sequelæ or a characteristic lesion, is abscess of the liver. Kartulis found it in about twenty-five per cent. of all cases that came to autopsy, and Craig found it in nine cases out of twenty-three, and it seems more than probable that a large proportion of cases have abscesses which do not develop enough to attract attention. I have used the common phrase "abscess of the liver," but this is in a way a misnomer. The contents of the collection is not a true pus. It is a fluid containing granular detritus, cells, and amebæ, but not a true collection of pus cells, and it is commonly without pyogenetic power.

*Mortality.*—No definite figures can be given for the mortality; often it is low, sometimes very high. In very few diseases do so many factors seem to be capable of affecting the result. The immediate prognosis in average cases is good, but the disease is exceedingly prone to reappear.

*Occurrence.*—It is wide-spread and frequent in occurrence in the tropics all over the world. It has been found in Russia, Italy, France, and other continental countries, as well as in the United States, but its prevalence and virulence in the tropics has led to its being quite commonly called "tropical dysentery." This is unquestionably a misnomer. It now seems likely that much of the severe and more rapidly fatal dysentery of the tropics is a distinct disease, due to the bacillus of Shiga.

Very little was heard of the disease by physicians in this country until the occupation of the Philippines. There the disease threatened to become a scourge, until the medical corps of the army took it energetically in hand, and, to their great credit be it said, have not only limited its occurrence but greatly reduced its mortality.

Cases have been reported from time to time in various parts of this country, but as yet very little is known of its distribution, and there is still a very general impression that its existence in this country is confined pretty closely to the more extreme Southern States.

While this is doubtless true to some extent (the water-borne character of the organism and its rapid loss of activity on slight chilling making it quite understandable), still it is far from the full truth.

The reports of the Johns Hopkins Hospital for the four years 1899–1902 show ten cases and other cases have been reported lately, and, while Baltimore is a maritime city with a cosmopolitan commerce, these are not all from foreign or southern localities, for McCrae stated in a discussion before the fifty-third meeting of the American Medical Association that some of these cases had not been outside of Maryland; and Walsh and Liberman stated at the same time that they had seen several cases in New York that had not been outside of the city. A case has been reported from Cincinnati. Dock has reported one from Michigan, and Nietert has reported nine from St. Louis. Now, there have been over 125,000 men returned from the Philippines during the last four years, and a very considerable proportion of these have had at some time attacks of dysentery of greater or less severity, so, as relapses are common, even after months, we will probably see and hear more of this disease in the near future.

Again, we are going to hear of it because we are going to look for it, and it is to emphasize the necessity for systematic examination of the stools in all dysenteric cases that I present the report which follows.

I do not know the history of the Cincinnati cases as to the place of exposure, but in two of the cases I have to report all chances of infection, except in this immediate vicinity, can be excluded, and the four cases are the first to be reported from Louisville or Kentucky. The ameba was found in the first of these cases by Assistant Surgeon Berry, who had previously seen cases in Galveston.

*A Word as to Technique.*—We have seen the ameba frequently in all the cases, but we have never found it in the absence of blood from the stools. Even when there are frequent, small, slimy stools, with some pain and tenesmus, we have not found it unless traces of blood were present. Other observers have found it in apparently normal stools, so that doubtless we are not as expert as we might be.

The stool should be caught in a well-warmed vessel, and the latter covered and stood in a basin of hot water. We have not used a warm stage. In a warm room, and on a previously warmed slide, the organism will live long enough for observation.

A portion of the stool, preferably one of the blood-stained flocculi, is picked up with an öse and placed upon the slide and the cover-glass pressed down firmly, so as to flatten the mass and spread out the film in a uniformly thin layer. A dry lens, such as a No. 7 Leitz, with a low eye-piece, gives the best results in finding the organism, but I like to put on an oil-immersion to study more closely. The appearance of the organism is very characteristic. It is very striking to see the clear ectosarc suddenly shoot out as if about to tear loose from the granular endosarc, which momentarily remains motionless, and then see the endosarc suddenly decide to flow out, as it were, into the new process. It is one of the easiest found and most striking of organisms.

The treatment of this disease may tax the physician's resources to the utmost in the matter of distressing symptoms, complications, and sequelæ, but the actual treatment of the disease itself can be covered by the well-known formula, "Remove the cause and put the part at rest." A local infection of, usually, an accessible location by a living agent, the treatment par excellence is the local application of agents which will destroy the organism. Determine as near as you can the probable site of the lesions in rectum, sigmoid, or further up in colon, and give frequent enemata, low or high, of suitable agents, preferably sulphate of soda or quinine, especially the latter. Pain and tenesmus must be controlled, of course, and I usually give tannigen if the stools show much evidence of coexisting catarrhal inflammation.

Neither of these cases can be classed as at all severe; one was quite mild, but it is because the apparently slight ailment masked a really serious affection, from the standpoint of prognosis, that is, that they are of value.

E., white, native of Kentucky, aged nineteen, well-built, well-nourished; admitted October 5, 1901. Was attacked by diarrhea about three weeks ago, shortly after returning from a trip down the river, extending to just below Memphis. Had cramps and tenesmus, and at end of first week noticed blood in his stools. Is not passing as much blood now as at first, and has only about six stools a day, mostly at night. Has cramps early each morning. Temperature and pulse normal. Was put on usual treatment for intestinal disorders and improved a little.

Stool examination was made on October 8 and large numbers of ameba found, also an actively motile organism, somewhat larger than the ameba and apparently flagellated. Ordered a gram of quinine in hot aqueous solution, as a high enema, twice daily, with ten drops of laudanum. The laudanum was only temporarily necessary. Patient had phimosis. He improved rapidly, but as he had phimosis he was circumcised, and discharge delayed until November 1. At that time his stools were of normal appearance, well formed. Careful examination failed to reveal the ameba.

He had what was apparently a relapse in the following February, 1902, but deserted as soon as he began to improve. He was again admitted, with a history of dysentery lasting some time, and a loss of almost thirty pounds in weight. He did not have many stools, five or six a day, but showed considerable prostration. Tannigen, orphol, bismuth, calomel, etc., were carefully administered, but while the stools diminished to one or two a day, and he gained flesh, they became frequent again if the diet was increased. Resource was then had to enemata containing one gram of bimuriate of quinine, with resulting rapid improvement and discharge on August 28. He came back February 10, 1903, with a history of a relapse beginning about a month previously, and stated that he had had one or two previously, since last admission, that had yielded readily to simple treatment. Present attack has gotten better and worse several times. Has from eight to ten actions a day, with blood and mucus in the stools. Is much thinner than when last seen, and has a troublesome cough. Stools showed ameba. Was given 0.3 gram of calomel, and after it had acted the high enemata of quinine were recommended. By the 16th the ameba had disappeared from the stools. On the 18th he had two normal actions and his diet was increased. On February 24 he was discharged. He was then having one perfectly natural stool a day.*

* This patient returned in May, 1903, with a slight relapse which, as before, yielded promptly to treatment.

W. J., white, aged thirty-nine, native of Indiana, admitted December 31, 1901. Following some headache and digestive disturbance for a couple of weeks, he was attacked during the night of the 28th with a violent choleraic attack. This was succeeded by a diarrhea—seven or eight stools in twenty-four hours, with tenesmus and cramps, and passage of blood and flaky mucus. Samples of stools examined by Assistant Surgeon Berry and by the writer, and a diagnosis of amebic dysentery made. His abdomen was markedly distended and tender, and his liver and spleen slightly enlarged. He was put on quinine enemata and also given small doses of naphtol, bismuth, and ipecac in capsules. He began to mend at once, and was discharged at his own request on the 9th of January.

C. M., white, native of Missouri, admitted to hospital December 8, 1902. Has always had good health; contracted diarrhea about a month ago, which lasted three weeks, not inconveniencing the patient much until cramping set in, and he went into the Cairo Marine Hospital, staying there five days, leaving apparently cured. About six or seven days ago the diarrhea again began, and he was having as high as fifteen or twenty actions in twenty-four hours, the stools consisting of mucus, no blood being noted. About ten o'clock on the night of December 6 he was suddenly seized with a severe pain in the right shoulder; pain seemed then, and seems now to radiate from the hypochondriac region of the same side. The pain is not felt when the patient lies on his back or left side, but any attempt to lie on the right side causes it to recur with great severity. Appetite entirely absent; has eaten scarcely anything for five days; the last two days has had a slight cough. The cough causes considerable pain—pain being felt in the epigastrium and right hypochondrium, and extending into the shoulder. The patient is a large, well-developed man, about six feet tall, very well proportioned; abdomen lank, rather retracted; respiration rather rapid, and somewhat costal in type; tongue moist and clean, but large and thick; heart sounds slightly increased in rapidity. On percussion, the liver is found to be enlarged, the dullness extending to the upper border of the fifth rib, and to an inch below the margin of the ribs. The left lobe of the liver can be easily palpated in the epigastric region. The left side of the abdomen is only slightly tender, the whole right side markedly so, being most marked near the margin of the ribs. Temperature 39°. Ordered rest, fluid diet, and one grain of tannigen every three hours. By the next morning the pain in the shoulder had largely disappeared, but a great deal of tenderness persisted in the hepatic.

Abundant amebæ were found in stools. His diarrhea rapidly yielded to treatment. His temperature vibrated between 39° and normal for a week and then settled down to normal. The pain, tenderness and engorgement in liver gradually diminished, and then disappeared, and on December 26 he was discharged at his own request. His blood was examined at the time of admission, and showed a well-marked leucocytosis. This was probably a case in which the formation of a liver abscess had begun but was arrested.

T. R., colored, aged seventeen, native of Tennessee, admitted to hospital December 18, 1902. Has always enjoyed good health until five months ago, when a diarrhea set in, which soon grew worse and became chronic, bowels moving frequently a dozen times a day, with passage of much blood and mucus. Was in the Evansville Marine Hospital for two weeks in August, and left apparently cured, but in October the disease reappeared, and soon became as bad as ever. During last week had frequently a dozen actions a day, and has rapidly lost flesh and strength, and has considerable pain in the left side of the abdomen. The patient is a small, yellow negro, usually weighing one hundred and fifty pounds; present weight, one hundred and twenty pounds or less. Abdomen rather distended, except over the descending colon, where tenderness is well marked. Faint murmur heard with first sound of the heart, and rhythm is the least bit irregular. Has not been farther away than Evansville or Louisville for over a year. The origin of infection therefore is local, either in this city or in this section of the country. On the 19th the stool was examined, and consisted of about two tablespoonfuls of bloody, gelatinous mucus, containing numerous ameba, motile and non-motile.

Put on usual treatment, as above, enemas being given through tube while patient was in knee-chest position. His improvement was rapid, but he was discharged December 18, before complete recovery. At that time his liver extended to about two inches below border of his ribs. In this case, as in the second one, the site of infection can be definitely limited to the immediate vicinity of Louisville.

Since the above was written, the first-mentioned case has again been under treatment, and I have seen two other cases, one being in hospital at the present time. It is therefore evident that the disease is a very common one in this locality.—*American Practitioner and News.*

---

### TREATMENT OF PUERPERAL ECLAMPSIA.*

While it is not within the scope of the subject of this paper to consider the many theories as to the etiology of puerperal eclampsia, it is essential that, before outlining a method of treatment, a brief statement of the recognized conditions existing and difficulties to be overcome should be made.

Without entering into details and only referring to them as a basis of treatment, the causes of puerperal eclampsia may be divided into predisposing and exciting. Among the first may be mentioned certain preexisting diseases, as of the heart, kidneys, liver, etc.; pregnancy in the unmarried, as noted by Wilger and Price; primiparity; multiple pregnancy and hydramnios. Especially does the hypersensitive and irritable condition of the pregnant woman render her liable to spasms. According to Windscheid, tetany and chorea rarely affect women except during pregnancy; a fact strongly emphasizing her susceptibility to nervous affections. The pregnant woman's physiologic condition predisposes her to puerperal eclampsia. Her blood, although increased in quantity, shows fewer red blood-cells and is burdened with excess of leucomaines and increased toxicity of serum. All her organs, especially heart, lungs, liver, kidneys and thyroid gland, must do extra work. With this knowledge, it behooves the family physician to keep her under constant surveillance, and to warn her that, with the first appearance of dangerous symptoms, she should seek medical advice.

*Read by B. M. Hypes, M.D., St. Louis, Mo., before the St. Louis Medical Society, May 30, 1903.

As to the exciting causes we must confess much ignorance. However, it may be stated as the consensus of opinion among obstetricians and pathologists of the present day, that puerperal eclampsia is caused by the retention in the blood of the pregnant woman of toxic material, originating from intestinal putrefaction and destructive metabolism, both in her own system and in that of the fetus (principally the latter); that she thereby suffers from a toxemia or auto-intoxication; that this retention of poisonous material is accompanied by insufficiency of the emunctory organs, especially of the kidneys and the liver and, according to H. Oliphant Nicholson, of the thyroid gland; that these organs are not primarily, but rather secondarily, affected, analogous to the pathologic conditions following scarlatina or diphtheria. The probable modus operandi in the production of the spasm is that these poisons, circulating in the blood, act as an irritant to the sympathetic nervous system, causing a contraction of the arterioles of the body and especially those of the brain. As a result of this contraction of the arterioles, the base of the brain suffers an acute anemia, while the large vessels of the cortex become engorged and press upon and irritate the cortical substance, thereby producing the spasm.

With this cursory review of the conditions to be met, we may more intelligently take up the treatment. This will be remedial, during a paroxysm, and prophylactic for threatened eclampsia.

In managing the conclusive state, the indications for treatment are to control the spasms, to relax the contracting arterioles, to deplete the engorged blood-vessels and to eliminate the exciting toxins.

To control the spasms, chloroform may be given to the surgical degree. This is the almost world-wide custom, but it has no curative effect, and when long continued is dangerous, as it favors broncho-pneumonia, increases the disintegration of red blood-corpuscles and fatty degeneration of vital organs. In the last two cases treated by the author, no anesthetic has been used, the patients having been managed during the spasm as in ordinary epileptic seizure. The inhalation of oxygen gas, as recommended by Professor W. Stroganoff, of St. Petersburg, seems a more rational treatment.

To further control the convulsions and to meet the indications for treatment outlined above, three remedies stand forth as pre-eminent; namely, morphine, chloral and veratrum viride. With or without an anesthetic, the author, in managing this disease, at once resorts to the use of veratrum viride. Fifteen drops of the fluid extract (preferably Squibbs') is administered hypodermically, and repeated in five- to ten-drop doses every half-hour until the pulse is reduced to sixty beats per minute. With a pulse thus lowered, eclamptic spasms are unknown. Veratrum viride, thus given, acts as a vaso-dilator, lessens the blood pressure, slows the pulse, lowers the temperature, induces diuresis and diaphoresis and assists in dilating the os uteri. Objection is made that it is a "too dangerous drug to use." The objection is probably without foundation, as it has been administered for the past fifty years in America, in large quantities and heroic doses, without a single fatality being reported. Should syncope threaten after its use, the reclining position, with opium and heart stimulants, will readily correct. This remedy has been employed by the author in ten cases, with one death. This one fatality ought not to be counted either for or against the remedy, as the patient was moribund when first seen and died within an hour.

In cases exhibiting plethora, engorged blood-vessels, edema of the lungs and other symptoms favoring its use, in addition to the administration of veratrum viride, the author resorts to that almost "lost art"—venesection. "Bloodletting forms the first step in the treatment of puerperal convulsions," says the late Dr. Lusk. It is adapted to all cases, save the anemic. Contrary to general teaching, a weak pulse is no contraindication. The majority of the author's cases have presented a rapid, weak or wiry pulse. The blood of the average woman, weighing 125 pounds, amounts to eight or nine pints. Bloodletting to the amount of one pint, the abstracted blood being replaced by an equal quantity of salt solution, will practically remove one fourth or one fifth of the toxic material, says Dr. Williams of Johns Hopkins University. By its use, the arterial tension, with accompanying arteriole contraction, will be lessened, the sympathetic irritation relieved and the normal functionating of the organs favored.

The general mortality from puerperal eclampsia is from 25 per cent. to 50 per cent. But statistics, gathered from our western physicians, who bleed freely—from sixteen to thirty ounces—show a mortality of only 7 per cent. Should the spasms return, venesection may be repeated; or, if contraindicated, other remedies may be used to which the venesection has made the patient more susceptible. In five of the author's ten cases, venesection was practiced.

Hydrate of chloral, either alone or combined with other remedies, has been used very successfully in treating this disease. Given in 30- to 60-grain doses with milk, per rectum, it not only aids in controlling the spasms, but lessens the annoying restlessness and irritability that follows.

But like chloroform, which it greatly resembles in action, it has its deleterious effects, and of late years has been abandoned by most practitioners for more successful remedies.

Morphine is the most generally recommended medicine, at the present time, for the control of puerperal convulsions. Following Veit's reports and recommendations, it is universally used in Germany and is becoming very popular in England and America. It is given in heroic doses, from ¼ to 1½ grains, hypodermically, repeated according to the restlessness or return of the spasms. However, its ill effects on some of the organs and the subsequent "locking up" of the secretions, are the objections to its administration. Although claimed as the "German treatment," morphine, in large doses, was first recommended by Dr. C. C. P. Clark, of Oswego, N. Y., in 1880 (American Journal Obst., 1880 and 1881).

At the present day, no surgeon would presume to undertake a trying operation without having at hand a prepared salt solution and the apparatus for the administration. Of paramount importance is the use of this remedy in puerperal eclampsia. Since Jardine, of Glasgow, reported (Scottish Med. and Surg. Jour., Oct., 1839), that by this treatment the mortality in the Glasgow maternity was reduced from 47 to 17 per cent., its application has increased in popularity until it has become almost universal.

The ease with which it can be administered—hypodermically, intravenously or per rectum—together with its almost immediately beneficial results, should commend its use to every practitioner. It acts as a stimulant to all the organs, especially to the heart and kidneys; it dilutes the toxins circulating in the blood, thereby rendering them less irritating to the nerve centers; it dilates the capillaries, thereby increasing diaphoresis and diuresis; and it promotes elimination through all the emunctories.

Another remedy of undoubted value in many cases is the "hot pack." It has a soothing, calming effect, principally by inducing diaphoresis and relieving the engorgement of the internal organs. Its use renders unnecessary the administration of that dangerous and depressing drug—pilocarpin.

Besides strychnia, digitalis and other stimulants, nitroglycerin is a remedy of proven value in eclampsia, as it both reduces arterial tension and vasomotor spasm, and, moreover, acts as a mild diruetic.

In order to eliminate toxins from the blood, catharsis, diaphoresis and diuresis should be encouraged. Croton oil, elaterium, calomel and thorough lavage of the bowels may be employed for the first; the wet pack for the second; and salt solution for the third. Strong medical diuresis should be avoided, as the kidney is already an overworked organ.

In cases where the foregoing measures fail and the spasms continue, obstetric interference may be necessary. It is a statistical fact that the convulsions cease in about 90 per cent. of the cases, as soon as the child is delivered. Hence, during labor we should hasten delivery by forceps or version, if the os is dilated or dilatable. In cases where labor has not begun or where the cervix presents marked resistance, then forcible dilatation by the hand or Bossi dilator, Duhrrsen's multiple incisions or cesarean section may be employed. However, statistics are, as yet, too meager to warrant obstetrical interference in all these persistent cases, and the majority of writers still prefer to rely upon the expectant treatment. In case of the death of the mother, with signs of a living child, rapid delivery through the parturient canal or cesarean section should be immediately performed.

Any paper on puerperal eclampsia would to-day be incomplete without reference to the articles recently published by H. Oliphant Nicholson (*Journal of Obstet. and Gyn. of the British Empire*, July, 1902), and by Prof. W. Stroganoff (*Obstetrics*, Vol. III., No. 2, February, 1901).

Dr. Nicholson believes that eclampsia depends upon inadequacy of the thyroid secretion. He says: "It is evident that the real significance of the pre-eclamptic stage is that it points to a breakdown of some part of the defensive mechanism. Furthermore, this breakdown is the result of some inadequacy of the thyroid and parathyroid glands, whereby the process of nitrogenous metabolism, instead of resulting in the formation of urea, ceases with the production of intermediate substances, which, when absorbed, excite the symptoms of a toxemia. In this way the degree of toxemia of pregnancy comes to be dependent, directly or indirectly, upon the quantity and activity of the thyroid secretion; the thyroid gland may therefore be given primary rôle in the causation of eclampsia." In accordance with this theory, he has treated the pre-eclamptic symptoms—albuminuria, edema, headache, vomiting, etc., with 5-grain doses of thyroid extract, given night and morning, with eminent success. Upon stoppage of the treatment, the symptoms would, in some cases, return; but they would again disappear upon the resumption of this remedy. He and others report favorable results from the use of the thyroid extract during the spasms. The theory and treatment are certainly worthy of consideration and investigation.

Professor Stroganoff makes the wonderful report of fifty-eight cases of eclampsia treated without a maternal death and with a fetal mortality of 11 per cent. He considers eclampsia an acute infectious disease, usually running a limited course which rarely exceeds twenty-four to forty-eight hours. He uses no chloroform or ether, but gives oxygen inhalation during an attack. After the first convulsion, he gives one-quarter grain morphin hypodermatically. With restlessness or spasm, repeats the dose in one hour. He then gives twenty to forty grains of chloral every five to ten hours to induce narcosis. With convulsions threatened or continuing he occasionally repeats the dose of morphin. He follows with quiet, fresh air, and occasional inhalation of oxygen, and when the case demands, dilates the os and delivers the child.

Contrary to the usual custom, I have left the prophylatic treatment to be considered last. I do this for the reason that I regard it more important. As before stated in this paper, the pathogenesis of this disease is in doubt. The best practitioners differ as to its therapeutics. But there can be no question as to the truth of the statement, that almost all of the cases of eclampsia may be prevented if proper prophylactic treatment be instituted. Remember that the defensive organs—the kidneys, liver and thyroid gland—are at fault. A large per cent. of the cases are associated with renal disease or insufficiency. This condition is accompanied almost invariably by albuminuria and other easily recognized symptoms. Not only is the quantity of urine lessened, but there is a deficiency in the normal amount of urea and other solids. This abnormal condition of the kidney, every practitioner of medicine should be able to diagnosticate. But how many of us take the trouble to examine the urine of the pregnant woman weekly, monthly, as we should?

Renal insufficiency ought to be early recognized and proper treatment at once inaugurated. Were this universally done, many women who now fill premature graves might be alive and happy with their families to-day. Puerperal eclampsia should be recognized as a preventable disease and as great care exercised by the physician to avert it as to prevent puerperal sepsis.

The prophylactic treatment will be hygienic and medicinal. With albuminuria, edema, persistent headache, disturbances of seeing and hearing, vomiting, epigastric pains and other prodromes of eclampsia, the patient should be put upon a strictly milk diet. Milk is almost a specific for the renal affections of pregnancy. Pinard, at a meeting of the Paris Academy of Medicine in 1893, said: "Thanks to this precaution, I have not observed a single case of eclampsia in more than 5,000 women admitted to the Bondelocque Lying-in Hospital since 1889."

Farinaceous food, white meats and fish may be allowed as symptoms improve. Not only should constipation be avoided, but with toxemic symptoms daily free catharsis by salines should be insisted upon. Thus, the liver which in some cases is no doubt the guilty organ, is kept active. By these means the toxemia is lessened without impoverishing the blood of its red corpuscles. In addition to milk diet, plenty of pure water should be taken to flush the kidneys. None but mild diuretics are permissible owing to the overworked condition of these organs. A warm bath should be given daily to promote diaphoresis. Flannel clothing should be worn to protect against chilling of the surface. Fresh air, sunshine, proper exercise and rest should always be insisted upon.

Medicinal treatment for toxemic symptoms will include cathartics, diuretics, diaphoretics, nerve and heart sedatives and, possibly, thyroid extract, and such general tonics as may be indicated. All these measures failing to give relief and the patient growing worse, the induction of abortion or premature labor will be justified.

### ADDENDUM.

Since writing the foregoing paper another case of puerperal eclampsia has come under the author's care which so typically illustrates the effect of the veratrum viride treatment that the following condensed notes are appended:

On May 5, 1903, was called to see Mrs. J., aged twenty-six years, pregnant, primipara, confinement expected three weeks hence. Questions revealed nothing abnormal in her previous history. Her mother stated that despite her pregnancy her general health had been good save "swelling of body," principally observed in limbs and face. Present illness began after eating a hearty supper the evening previous, with severe pain in the pit of the stomach (Olshausen's pathognomonic sign of eclampsia), vomiting, headache and marked restlessness. Symptoms continued in aggravated form until spasms began about 12 A.M., May 5. These spasms continued at frequent intervals followed by insensibility, until the author arrived at 2:45 A.M. He found her in a profound stupor from which it was impossible to arouse her. Pulse 130, weak and thready; temperature 100.5° F.; deglutition impossible; vaginal examination revealed os uteri and cervix normal to last month of gestation, but no dilatation or other signs of the onset of labor. At 3 A.M. patient was again siezed with a most violent spasm. Medicines were sent for and a high enema given. At 3:25 A.M. fifteen (15) drops of Squibbs's fluid extract of veratrum viride were administered hypodermatically. At 4:25 pulse 112; temperature 100° F.; veratrum (15 drops) repeated hypodermatically.

4:35 final spasm much milder.

4:55 pulse 90, soft and full.

5:15 pulse 70, temperature 99°.

6:20 pulse 60, temperature 99°.

8:45 pulse 72, patient restless, signs of beginning of labor. Ten drops of veratrum given.

12:45 P.M. living child delivered, easy labor, breech presentation.

1:15 P.M. placenta expelled with considerable blood.

Subsequent treatment—calomel, croton oil, salines and high enemata for catharsis. Lithia citras and "Mountain Valley Spring water" (Ark.) were given for diuresis.

Examination of urine drawn by catheter showed a large quantity of albumins, blood-corpuscles, hyaline and epithelial casts; solids of urine diminished.

It was forty-eight hours after birth of child before patient recovered consciousness sufficiently to recognize members of her family. Otherwise puerperium was normal for both mother and child.

Four weeks later, frequent examinations of urine made since confinement all show the patient to be suffering with chronic nephritis, though she looks and feels well.—*St. Louis Medical Review.*

---

### THE SENSATIONS OF DROWNING.

Dr. James A. Lowson, in the *Edinburg Medical Journal* for January, gives a graphic account of his experience in drowning. He says:

"After what appeared to be ten or fifteen seconds, the effort of inspiration could no longer be restrained, and pressure on the chest began to develop. Probably the most striking thing to remember at this period of time was the great pain produced in the chest, which increased at every effort of expiration and inspiration; it seemed as if one were in a vise, which was gradually being screwed up tight until it felt as if the sternum and spinal columns must break. Many years ago, my old teacher, Sir Henry Littlejohn, used to describe how painless and easy a death by drowning was—'like falling about in beautiful green fields in early summer'; this flashed across my brain at the time, and I said to myself, 'Poor old devil, Littlejohn—scarcely so accurate that time.' The 'gulping' process became more frequent for about ten efforts, and hope was then extinguished. . . . The pressure after these ten rapid 'gulps' seemed unbearable, but gradually the pain seemed to ease up as the carbonic acid was accumulating in the blood. At the same time the efforts at inspiration with their accompanying 'gulps' of water occurred at longer and longer intervals. My mental condition was now such that I appeared to be in a pleasant dream, although I had enough will-power to think of friends at home, and still retain vivid recollections of the clearness of the sight of the Grampians, familiar to me in boyhood, which was brought to my view. Before finally losing consciousness, the chest pain had completely disappeared and sensation was actually pleasant. What time I had then passed in the water I cannot possibly say—but I should think about two minutes; I was greatly handicapped below water by the previous exertion in getting on deck, and then by the stunning blow on the head, with the result that instead of going down after the full inspiration there was actually very little more than residual air in the lungs. Then the useless attempt to reach the surface would further reduce the time necessary to produce unconsciousness. What happened when inspiration was attempted was that the mouth was immediately filled with water, and, the epiglottis closing or closed down on the larynx, the act of swallowing at once occurred. I think the only time the epiglottis was not close down was during the short expirations which took place after every attempt at inspiration.

"The article on drowning in the Encyclopedia Britannica says: 'The drowned individual struggles to reach the surface of the water in his efforts to respire—as he does so he draws water into his windpipe which provokes cough.' I cannot see how it is possible for a man under water to cough, and I do not believe that any water will get into the trachea until after unconsciousness comes on. I have made post-mortem examination of scores of drowned bodies in Hong Kong, and 'froth in the trachea' is anything but a constant sign. Where efforts at artificial respiration have been made, one would expect this frothy condition, or if in drowning they had come to the surface once or twice. Captain Marryat's experience of drowning was that the sensations were rather pleasant than otherwise, and Sir Henry Littlejohn seems to have almost used Captain Marryat's words. They are both wrong—Marryat probably forgot about some of his sensations.

"To go on with the narrative: consciousness returned, and I found myself at the surface of the water, and managed to get about a dozen good inspirations. A hurried glance showed me the land about 400 yards distant, and I proceeded to utilize first a bale of silk and then a long wooden plank to assist me to the shore. These and the life-belt were of the greatest use also in saving my body from being dashed about on the reef in the tempestuous sea. As it was, feet, knees and the regions of the anterior superior iliac spines were considerably lacerated. On landing and getting behind a sheltering rock, no effort was required to produce copious emesis. I do not think that much, if any, water could have got down the trachea. I gave it the chance to run out by gravity, while vomiting. It would be interesting to make sure at what stage the epiglottis begins to refuse work. The possibility of pneumonia or bronchitis occurring was soon forgotten in the further exigencies of the situation."

---

### MEDICAL TEACHING AND MEDICAL RESEARCH, AND THE INCREASING OPPORTUNITIES IN AMERICA TO ENTER ITS CAREER.

Only a few years ago this country offered but one distinctly medical career to the student of medicine, namely, the practice of medicine in some form or other.

Twenty years ago there was but an insignificant number of salaried medical professorships; assistants were not necessary because the teaching was done almost wholly by didactic lectures; very little attention indeed was given to research in the medical schools, and endowments for medical research existed only in the dreams of the visionary. But within a remarkably short time a most marvelous change has taken place.

The elevation of the standards of medical education, the introduction of laboratory methods in medical teaching, the fostering influence of our best universities, and the steadily increasing endowments of medical teaching and investigation are some of the most important elements in the growth that has led up to the present period of promise, the blooming period of medicine in America.

Rapid and healthy development of this nature cannot but be associated with an increasingly diversified differentiation of function, and so there is now larger opportunity than ever before for successful careers as medical teachers and investigators to the exclusion of the actual practice of medicine.

Many of our best universities now offer practically the same inducements to properly qualified young men to join the departments of Anatomy, Physiology, Pathology, and Bacteriology as they do in the case of other university departments, let us say, History, Physics, Chemistry, and Zoölogy. In not a few instances positions in the medical departments just named are filled with success by men who have specialized in some one of these lines without having taken the regular courses in medicine. This fact is cited here simply to show that these fields offer welcome opportunities to qualified aspirants for academic careers.

In the more purely clinical branches the conditions as yet are somewhat different. Professors and instructors in these branches rarely, if ever, give their time and energy undividedly to teaching and investigation, but combine this work with the arduous duties of private practice. But even in these branches, related so intimately to the practical work of the physician, indications point to new developments that before long may culminate in the establishment in some of our universities of departments of medicine, of surgery, and of obstetrics upon the same general basis as other university departments. Necessarily this must lead to the foundation of university hospitals as well as to the creation of a large number of positions to be filled by specially trained men who are willing to devote themselves wholly to the teaching of the clinical branches and to the investigation of the many important problems that await solution in these fields.

We believe, too, that medicine in the universities is receiving its full share of fellowships, scholarships, and the like with which to encourage scientific work by students and young graduates.

Then again the recent endowments of independent institutions for medical research will offer especially desirable opportunities for those whose ability and training fit them for the highest order of investigative work. The establishment, too, of research professorships in the universities may be found to prove advantageous in order to advance to greater opportunity the man of genius sure to appear from time to time as the number of men engaged in productive work in the medical sciences increases.

Attention may be directed also to the fact that government and municipal services are demanding an increasing number of specially trained men to carry on their work, much of which is of the nature of research in various lines. The health departments of our larger cities need men trained in sanitary science; the Philippine civil government has established laboratories in which active investigation is carried on in the rich field of tropical diseases.

The managers of many of our State hospitals for the insane are beginning to realize that the surest antidote to the fossilizing influence of routine and institutionalism is to be found in the reorganization of the medical work upon a scientific basis so as to attract to this service men of scientific interests and training.

More than enough has been said to make good the statement that medicine in America now offers much more by the way of opportunity for successful careers than only the chance to enter the overcrowded ranks of the practitioner. It will ever be the hope of all who are interested in the progress of medical science among us that the opportunities for productive scholarships may increase in scope and in number, and that they may be made so attractive as to draw into definite careers along the lines pointed out in the foregoing the finest types of our medical students.—*University Record.*

THE JOURNAL OF THE AMERICAN MEDICAL ASSOCIATION.

Our distinguished friend, Dr. J. C. Culbertson, editor of the Cincinnati *Lancet Clinic*, in a recent editorial in his journal comments in an interesting way on a few incidents that he noticed at the banquet given to the members of the American Medical Editors' Association in New Orleans last week. Among other significant things, this distinguished gentleman said:

"The writer is tempted to make mention of an incident, perhaps excusable and perhaps not. The incident thought of was the absence of the editor of the *Association Journal* or of a representative. A self-assumed representative, in responding to a toast, took occasion, foreign to his subject, to thank the independent medical journalists for the aid and assistance rendered from time to time by them to the *Association Journal,* and asked for a continuance of good-will and fraternal fellowship. This expression was received with solemn silence, and why should it not be so? There was certainly no concert of action on the part of the independent journalists on the matter, but there was evidently a common feeling of dissatisfaction with the course of that publication. Evidently, a line has been drawn and the foundations of a wall erected which has for its intent and purpose a future antagonism of the *Association Journal.* There was not much outspoken feeling expressed, but mutterings and murmurings of discontent passed around from one to another.

The sentiment referred to above is indirectly but ingeniously and forcibly expressed. This dissatisfied feeling that he refers to among the editors and publishers of the different journals is increasing every year and it is only a question of time when a "concert of action" will prevail. This is only as it should be. It is perfectly natural and perfectly right. If the success of a great organization like the American Medical Association is dependent upon its department of medical advertising to keep it strong and to keep it prosperous, let it weaken and let the interest in it wane.

In England there is a precedent for the policy adopted by our national association, and it is well enough for us in this country to study this policy and what it has led to there. I think that it is putting it mildly when I say that it has crippled medical journal enterprises in that country. I believe that it would be nearer the truth for me to say that it has run a great many of them out of business, and has kept many more from entering it. The British Medical Association, or its official organ, controls three-fifths of the medical journal advertising business in England. Things drifted to this point there many years ago, probably on account of the great strength of the British Medical Association and the evident weakness and want of business foresight on the part of the medical editors and publishers in that country. The American medical profession will not be so wanting in business judgment as to let their personal business interests sag without a formidable protest.

As a member of the American Association I don't believe that it would be amiss or improper in any sense or an injustice to any one to say that the Journal of the American Medical Association should not be the greatest advertising medium for proprietary medicines in this country, but should alone boast of its scientific superiority. As long as this policy of the journal prevails evidences of prejudice against it will be in evidence. When they make the advertising of the proprietary medicines an insignificant feature of the journal, or better still, do away with it altogether, then, and not until then, will the name of the association journal create a different murmur from that described by Dr. Culbertson, or cause the applause that it in many ways deserves. I believe that a policy of this kind would be a help to the journal's great editor and his proficient assistants and enable them to do better and more acceptable work.—*Charlotte Medical Journal.*

---

### RECENT ADVANCES IN THERAPEUTICS.

An unassimilated and defectively translated German physician, upon locating in Chicago, immediately published a text-book. Whooping-cough he discussed as follows: "Pertussis; Tussis Convulsiva. By this sickness we cannot earn much honor, for when we do something it takes six weeks; and if not, it takes the same time." Conceding the justification for this pessimistic view, there is hardly reason to extend it to all diseases, as physicians occasionally do in an access of melancholy.

From the depressing therapeutic nihilism of the past few years there has been a healthy reaction. Drugs are no longer prescribed in such lavish profusion and in heterogeneous combination as formerly, but the simple successor of the shotgun prescription counts for more and more as the years of medical knowledge.

And, in any event, since the term therapeutics is not limited to the use of drugs, but includes the application of all agencies for the cure, relief or prevention of disease, the profession can point with pride to its recent record. Most especially in the effective prevention of certain virulent epidemics is there cause for rejoicing. Progress in therapeutics is of necessity gradual, and an occasional inventory is necessary to mark the advance. Such a review is furnished by Satterthwaite's address before the American Therapeutic Society (*Medical News*, May 16).

Satterthwaite regards the suppression of yellow fever in Havana as the most brilliant therapeutic achievement of recent years. After an eight-months' crusade against the mosquito, the disease disappeared entirely from that city. In 1900 there were 300 deaths from yellow fever; in 1901 not a single death occurred although there were 40,000 non-immunes in the city. The death-rate was reduced from 45 to 22.11 per thousand.

In the Philippines, although cholera continues to slay the natives at an appalling mortality rate—60 per cent. of those attacked—the spread of the disease among the American soldiers has been effectively restricted by quarantine method. In the drug treatment of cholera some success is reported by Dr. Freer with benzoylacetylperoxid. Guaiacol carbonate and calomel have also yielded excellent results.

In the prevention of plague, inoculation with the Haffkine serum has reduced the number attacked from 85 to 77 per cent. The Yersin-Roux serum, according to the French commission, reduced the mortality from 70 to 14 per cent.

The death-rate from tuberculosis is steadily diminishing. This has been accomplished mainly by improved hygienic measures and sanatoria.

The wonderful results from the use of diphtheria antitoxin need not be dwelt upon. It is only fair to say, however, that the tremendously diminished death-rate is by some attributed to lessened virulence of the bacterium.

In chronic diseases of the heart, hydrotherapy, exercise, diet, general tonics and purgatives are assuming prominence, while the nitrites are more frequently substituted for digitalis and strophanthus. Preparations of the suprarenal capsule are now used very generally in appropriate conditions, with good results.

In conclusion, Dr. Satterthwaite is encouraged to believe that eventually America will control alien scourges as successfully as it dominates alien races.—*St. Louis Medical Review.*

---

### THE REPORT OF A CASE OF POLYMAZIA.

BY JAMES M. CRAIGHILL, M.D., OF BALTIMORE.

The patient in this instance was a colored woman, who came into my hospital ward on the 2d of March, 1903; aged forty-five; married; was suffering with a specific ulcer on the tonsil. She was of very low mental caliber, and when I questioned her as to the number of children she had she could not remember whether it was seven or eight. In the course of the usual physical examination I found under each arm a tumor about the size of a lemon and the tip surrounded by an areola, which felt like ordinary mammary gland tissue. During her nursing period she said they contained milk which exuded from them freely and at that time they would be three or four times the size they were when I saw her. There is no history of a similar condition in any of her family.

A case has been reported where the two extra breasts were as large as the normal ones and situated on the upper part of the abdomen. A supplemental nipple, a short distance from the normal ones, is a more frequent anomaly, several cases of this kind having been seen. In one family the condition was hereditary. There were originally in man five mammary glands, two at the center of the thorax, two in the axillary space and one about the umbilicus. Sanderson saw five nipples in these different situations in a man. Some writers think that the condition indicates a reversion to a lower type than man. This woman was, as I have said, of a very low type of the human species.

According to Gould and Pyle, polymazia is much more frequent than is generally supposed, seventy cases in females and twenty-two in males having been collected. The general site is the natural breast, then in the axillary spaces, and then in various parts of the body. They may be located in the vulva. A case is reported of a man with six nipples, three on each side, as in a bitch or sow. The unfortunate Anne Boleyn, wife of Henry VIII., had six toes and six fingers and an extra breast. We know that curious cases of lactation in old women occur, brought about by giving their breasts to children to sooth them. A case is reported of a grandmother of sixty-eight who nursed numerous grandchildren in succession. The condition is not altogether confined to the female sex. A noted case occurred in Baltimore, where a negro man had been wet-nurse to his mistress's children, and the case was exhibited at the University of Maryland many years ago. Cases have also been reported of milk in the breasts of very young children.—*Gaillard's Medical Journal.*

---

### DETERIORATION OF THE AMERICAN NEGRO.

Notwithstanding the heroic efforts and marvelous success of certain American negroes in demonstrating the capacity of their race for the highest attainments of man, and in providing opportunity and incitement for such efforts on the part of others, the general situation of the colored race emancipated from the control that was for the time being their best possible friend, is unspeakably deplorable. We have heretofore reprinted testimonies to this effect on physiological lines, from some of the best medical and hospital authorities in the South. The question what can be done to stem the downward course of this great element in our national population, seems to be one of the leading questions of the day.

We learn that in the Atlanta National Conference of Charities and Corrections the statement was made by experts that the negro race is in danger of being destroyed by insanity. The discussion was brought about by a paper read by Dr. Searcy, Superintendent of the Alabama Hospital for the Insane, who claimed that his experience showed that insanity was increasing among the negroes at an alarming rate.

Dr. Searcy declared that lineal deterioration was largely responsible for the tremendous increase of insanity among the negroes, there being *thirteen times as many insane negroes now as at the close of the war.* Dr. Searcy said:

"Before the war the negroes were of the most splendid physique and possessed of the finest health of any race in the world. This was due to the fact that the negroes were forced by their masters and their mistresses to be cleanly and to live a sanitary life."

"But conditions have changed. With freedom thrust upon them the negroes were left to hustle for themselves. They had no money and were forced to go scantily clad and live upon food cast aside by the white people. Without any one to direct them they became filthy, and the conditions under which they have lived have been unsanitary. As an evidence of how they live I call your attention to the fact that only recently a house was raided by the police in which seventeen negroes, men, women and children, were found living together within one small room, without a single bedstead and only a handful of dirty, unsanitary bedclothing to sleep upon. No wonder their health has been impaired, and no wonder there has been lineal deterioration.

"Insanity has been the result, and to-day this affection among the negro race is increasing with appalling rapidity. What are we going to do about it? Send them to hospitals after they have gone insane and let them die? If there is any such thing as genuine philanthropy, if there is truly a disinterested and unselfish desire on the part of the delegates to this conference and of people all over the country, let us take steps to change the conditions under which the negroes are living and thus avert the increase of insanity, which is making such inroads upon the negro race."

Other Southern physicians agreed with Dr. Searcy.—*N. Y. Times Report.*

